# Risk Factors for Children's Receptive Vocabulary Development from Four to Eight Years in the Longitudinal Study of Australian Children

**DOI:** 10.1371/journal.pone.0073046

**Published:** 2013-09-11

**Authors:** Catherine L. Taylor, Daniel Christensen, David Lawrence, Francis Mitrou, Stephen R. Zubrick

**Affiliations:** 1 Centre for Child Health Research, The University of Western Australia, Perth, Western Australia, Australia; 2 Population Sciences Division, Telethon Institute for Child Health Research, Perth, Western Australia, Australia; University of Leicester, United Kingdom

## Abstract

Receptive vocabulary develops rapidly in early childhood and builds the foundation for language acquisition and literacy. Variation in receptive vocabulary ability is associated with variation in children's school achievement, and low receptive vocabulary ability is a risk factor for under-achievement at school. In this study, bivariate and multivariate growth curve modelling was used to estimate trajectories of receptive vocabulary development in relation to a wide range of candidate child, maternal and family level influences on receptive vocabulary development from 4–8 years. The study sample comprised 4332 children from the first nationally representative Longitudinal Study of Australian Children (LSAC). Predictors were modeled as risk variables with the lowest level of risk as the reference category. In the multivariate model, risks for receptive vocabulary delay at 4 years, in order of magnitude, were: Maternal Non- English Speaking Background (NESB), low school readiness, child not read to at home, four or more siblings, low family income, low birthweight, low maternal education, maternal mental health distress, low maternal parenting consistency, and high child temperament reactivity. None of these risks were associated with a lower rate of growth from 4–8 years. Instead, maternal NESB, low school readiness and maternal mental health distress were associated with a higher rate of growth, although not sufficient to close the receptive vocabulary gap for children with and without these risks at 8 years. Socio-economic area disadvantage, was not a risk for low receptive vocabulary ability at 4 years but was the only risk associated with a lower rate of growth in receptive vocabulary ability. At 8 years, the gap between children with and without socio-economic area disadvantage was equivalent to eight months of receptive vocabulary growth. These results are consistent with other studies that have shown that social gradients in children's developmental outcomes increase over time.

## Introduction

Our uniquely human capacity for language is one of the most important developmental accomplishments of childhood. Language enables literacy, education, and employment [1], [2], [3], [4], [5], [6]and is one of the major pathways that supports human capability formation [7]. School is regarded as the single best investment in human capability formation in individuals and populations [8], however, the foundations for success at school are built early. Increasingly, early childhood (birth to 4 years) is the focus of global and national public policy frameworks for human capability expansion[9], [10], [11].

It is well known that language acquisition is not robust for all children and that disparities in language acquisition emerge early and foretell persistent low levels of language abilities. Longitudinal studies have shown a consistent pattern of early emergence of disparities in language acquisition that persist over time and influence disparities in later educational achievement [2], [4], [6], [12], [13]. Results from the first Australian national census of child development conducted in 2009 illustrated the extent of disparities in language abilities in children in the first year of formal school at 5 years. The results of the 2009 Australian Early Development Index (AEDI) showed that 23% of children were vulnerable in language and cognitive skills and that 25% were vulnerable in communication skills [14], [15].

Receptive language ability is a dimension of language that emerges in the first year of life and tracks strongly to children's literacy abilities [16], [17]. Our receptive vocabulary comprises words we know. Children show reliably that they understand the meaning of words they hear from around 8 months, well before they say their first words around their first birthday [18], [19], [20]. From onset in infancy, receptive vocabulary develops rapidly in the preschool and school years, from around 200 words in the second year [21], to 20,000 words at 8 years [22]. These cross-sectional studies of receptive vocabulary abilities have shown striking individual differences in receptive vocabulary size at different ages.

Population studies that have identified children in the low range of language ability at an early age and followed them up at a later age have revealed a mixed picture of persistent low language ability for some children and catch-up for others [23], [24]. These studies point to differences in the rate of language growth between ages, and prompt the question, what factors influence language growth between ages?

Guided by bioecological [25]and transactional [26] models of child development, studies that have used direct observation of parent-child interaction have necessarily investigated the influence of a restricted set of candidate predictors on receptive vocabulary development. This is because data collection, using direct observation, is so resource intensive [27]. Large-scale prospective population level cohort studies permit investigation of a comprehensive range of child, maternal and family level influences on receptive vocabulary development. For resource reasons, proxy, rather than direct measures of home environmental support for language learning are used in large-scale studies such as LSAC [28].

### Child, Maternal and Family Influences on Children's Language Acquisition in LSAC

LSAC is an ongoing life course epidemiological study designed to answer questions about how a child's individual characteristics interact with parental, family, community and school characteristics to shape development. The overarching aim of LSAC is to provide a contemporary evidence base for policy, prevention and intervention initiatives in Australia. Data collection for the study is led by a consortium of expert Australian researchers [29]. Guided by a bioecological model of child development [25], data are collected on child, parental, family, community and school characteristics that influence children's development at different ages (i.e., a developmental pathways approach [26], [30]. The measurement framework is comparable to indicator frameworks used internationally [31]. Indicator frameworks group variables that influence child development into key domains. For example, time, income, human capital, psychological capital and social capital [32]. This has produced a comprehensive (and expanding) set of independent variables that researchers can select from, to model in relation to specific developmental outcomes, which in this study, is language development [29].

The focus of this study is on factors that influence variation in receptive vocabulary abilities in the preschool and school years in an unselected population level sample. Population level research in language acquisition is rare, meaning there were few precedents for selecting candidate predictors of variation in language acquisition for this study. We selected child, maternal and family variables for our models based on two criteria: (1) Evidence of an independent association with English language abilities in an unselected population level sample of preschool and school age children; or (2) conceptual relevance to language abilities, in the absence of empirical evidence.

This study permitted the rare opportunity to bring together all of these predictors in the same models and to multivariately adjust for the independent influence of these predictors on receptive vocabulary growth from 4–8 years, in an unselected population level sample.

#### Child characteristics

The child characteristics in our models were gender, birthweight, ethnicity, temperament, school readiness and ear infections. Male gender, low birthweight [23], and minority race [28] have been identified as independent risk factors for low receptive language abilities. Temperament was included in our models because of the plausible relationship between a child's temperament characteristics, parent-child interaction and word learning. Low persistence is a candidate risk factor for low receptive vocabulary ability, based on evidence that low persistence was more prevalent in four-year-old children with low language abilities, relative to children with typical language abilities [33]. We included school readiness in our models because disparities in foundational literacy and numeracy skills have been shown to emerge as early as 22 months and to persist through the school years and into adulthood, especially for children with low socioeconomic status [1], [13]. Ear infections were included in the models, although surprisingly, this risk factor has not previously been examined in an unselected population level sample of preschool or school age children. What is known, is that in the LSAC sample, ear infections at four years were associated with a four-fold risk for hearing impairment at eight years [34]; and children with hearing impairment had lower levels of receptive vocabulary ability compared to children with normal hearing [35].

#### Maternal characteristics

We modeled maternal characteristics from three key resource domains for child development; human capital, psychological capital and time [36]. Our measures of human capital were age at the birth of the child, education and parenting. Our measures of psychological capital were mental health distress, problematic alcohol use and smoking. Our measure of time was hours of paid employment.

The most extensively studied variable is maternal education, with low levels of maternal education consistently associated with low receptive language ability [23], [37]. Parenting was included in our model as proximal measures [25] of human capital. High levels of positive parent-child interactions are associated with high levels of receptive vocabulary ability [38]. Maternal age was included in the model as an additional measure of human capital. Evidence from the only study, to examine the associations between maternal age, education and low receptive language ability, showed that low levels of education were associated with low receptive vocabulary ability and that young maternal age was not an independent risk for low receptive vocabulary ability. The same study included maternal mental health distress as a measure of psychological capital and reported no association with low receptive language ability [23]. We included mental health distress in our model as well as two health risk behaviors, problematic alcohol use and smoking, that are associated with mental health distress [39]. Full-time maternal employment has been associated with lower language abilities in preschool age children although the effect size was negligible [40]

#### Family characteristics

The family characteristics in our models were: Non-English Speaking Background (NESB), family structure, sibship size, income, health care card, financial hardship, socioeconomic disadvantage, reading to the study child, playgroup and child care. Four measures of socioeconomic status (SES) were included in our model: Income, health care card, financial hardship and socioeconomic disadvantage. NESB, family structure, sibship size, SES and reading to the study child are indicators of family resources for language development. Playgroup attendance and hours in child care are measures of the child's wider ecology, that is contexts other than the family. NESB and socioeconomic disadvantage are independent risk factors for low receptive language abilities [23], [38]. Single-parent family structure has also been associated with lower receptive vocabulary ability, relative to children in nuclear families [38]. Being an only child is associated with higher verbal abilities compared to children lower in the birth order [41] and large sibship size is associated with lower verbal and nonverbal abilities and lower academic achievement [42]. Receptive vocabulary abilities vary with SES, with lower levels of SES associated with lower receptive vocabulary abilities. This pattern is found when individual [23] and composite [28] SES measures are used. Book reading is a proximal measure of the home learning environment and is associated with variation in language abilities and foundational literacy and numeracy abilities [43] [38]. Playgroup attendance was included based on prior evidence of an association between attending playgroup and better learning (including verbal) outcomes for children growing up in disadvantaged families [44]. Hours in child care was included in our model because a high proportion of children (28%) in our sample spent more than 4 hours a day in non-parental care, during the working week. It was of interest to determine whether the amount of hours in non-parental care was an independent influence on children's receptive language abilities, over and above the other child, maternal and family characteristics in our models. Prior evidence has suggested that hours spent in non-parental care was not associated with variation in children's language abilities [45]. The present study investigated receptive vocabulary growth from 4–8 years in the nationally representative Longitudinal Study of Australian Children (LSAC). The specific aims were to (1) identify child, maternal and family risks for low receptive vocabulary ability at 4 years and (2) identify child, maternal and family risks for growth in receptive vocabulary ability from 4–8 years. Of particular interest, was whether or not the risks for a low intercept at 4 years were associated with a lower rate of growth from 4–8 years.

## Methods

### Ethics Statement

The Longitudinal Study of Australian Children (LSAC) is conducted in a partnership between the Department of Families, Housing, Community Services and Indigenous Affairs (FaHCSIA), the Australian Institute of Family Studies (AIFS) and the Australian Bureau of Statistics (ABS). The study has ethics approval from the Australian Institute of Family Studies Ethics Committee. The Ethics Committee is registered with the Australian Health Ethics Committee, a subcommittee of the National Health and Medical Research Council (NHMRC). As the study children were all minors at the time these data were collected, written informed consent was obtained from the caregiver on behalf of each of the study children. The signed consent forms are retained by the field agency (ABS).

### Access and Use of LSAC Data

LSAC data are publicly available. Researchers can apply to the Commonwealth of Australia Department of Families, Housing, Community Services and Indigenous Affairs (FaHCSIA) for permission to access and use Longitudinal Study of Australian Children (LSAC) data (FaHCSIA website. Available: http://www.fahcsia.gov.au/our-responsibilities/families-and-children/programs-services/growing-up-in-australia-the-longitudinal-study-of-australian-children-lsac. Accessed 2013 Aug 2).

### Study Design

The Longitudinal Study of Australian Children (LSAC) is a national longitudinal study that commenced in 2004. The study uses a cross-sequential design of biennial face-to-face visits with the family and study child. In this study we used the first three waves of the child cohort which comprised 4,983 children at wave 1 (age 4–5 years), 4,464 children at wave 2 (age 6–7 years) and 4,331 children at wave 3 (age 8–9 years).

The LSAC sampling frame was extracted from the Medicare Australia enrolment database, which was validated to ensure coverage of Australian children within the target age-range. The initial study sample was aimed at being representative of Australian children within the selected age cohort, proportional to the regional distribution of children in the Australian population. An initial sample size of 5,000 was chosen as to ensure there would still be a sufficient sample for detailed analysis after attrition over the number of years of the longitudinal study.

The study entailed a two-stage clustered design, first selecting postcodes then children within postcodes. Stratification was used to ensure proportional geographic representation for states/territories and capital city statistical division/rest of state areas. Cluster sampling was utilised because it provides a cost effective way to conduct face-to-face interviews, as well as an opportunity to collect and analyse community-level effects. Postcodes were selected with probability proportional to size selection where possible, and with equal probability for small population postcodes. Children were selected from 311 postcodes [46], [47].

Analyses show that the initial sample was broadly representative of the general Australian population when compared with 2001 Census data, but slightly under-representative of families who were single-parent, non-English speaking and living in rental properties. Attrition somewhat increased these biases. For example, the overall attrition rate between Waves 1 and 3 was 13%, but children with mothers classified as Non-English speaking background decreased from 15.7% at Wave 1 to 13.8% at Wave 3, an attrition rate of 23%. The proportion of mothers who had a year 11 or less education as at Wave 1 decreased from 39.2% at Wave 1 to 36.5% at Wave 3, an attrition rate of 19% [48]. Such attrition is typical in longitudinal studies. The growth curve modelling used in this study utilises maximum likelihood estimation; this technique makes full use of available data, which minimises the effects of item-non response within the study although does not fully adjust for missing data.

### Measures

A bioecological model of child development [25], [49] guided the selection of measures for LSAC. The conceptual model posited multiple domains of proximal and distal influences on child development. Among these domains are characteristics related to the child, the mother, and the family home environment. Many of the measures are benchmarked against Australian census collections while still others are referenced to large scale Australian and international child development studies. For ease of summary the measures used in the growth modeling for this study are classified into the above domains and with respect to their function (i.e., response, time and predictors).

#### Response variable

The response variable was the children's performance on the Adapted Peabody Picture Vocabulary Test-III (PPVT-III). The children were assessed by the interviewer with a number of tests, including the Adapted PPVT-III, a test of receptive vocabulary designed for the LSAC study [50]. The Adapted PPVT-III is a shortened version of the PPVT–III [51]. The Adapted PPVT-III was administered directly to each child during the home interview. For each word presented, the child was shown a card containing four pictures and was asked to point to the picture corresponding to the word (e.g., “Show me wrapping”). Scaled scores for the Adapted PPVT-III were used in all analyses. While the full details of the development of this shortened version are available elsewhere, briefly the Wave 1 calibration entailed an independently drawn sample of 215 children aged from 41 to 66 months (mean = 54.7 months) who were given the full PPVT-III with test administrators following standard procedures. After testing, a one-parameter (Rasch) item response model was fitted to the data, which consisted of correct and incorrect responses. The person separation reliability was 0.88. After determining the ‘best’ 40 items for use in a shortened version, the remaining items were then fit again to a one-parameter item response model; the person separation reliability was 0.78. In this way a short version of the PPVT-III was developed suitable for administration to the child in a household survey. The Pearson product-moment correlation between the full PPVT-III and the Adapted PPVT-III was 0.93 for all children [50]. This procedure was carried out at each Wave with appropriately drawn calibration samples. Thus the Adapted PPVT scores used in this report reflect a continuous range of ability. Table 1 contains the median ages and age ranges in months for the study children at each Wave along with the mean Adapted PPVT-III Rasch scaled score with SDs and associated ranges. For economy of expression, the Adapted PPVT-III is referred to as the PPVT.

**Table 1:** Children's ages and PPVT scores by longitudinal wave and sample size.

Wave (N)	Child's age in months			Adapted PPVT-III scores	
	Median	Range	Mean	SD	Range
1 (4983)	57	51–67	65	6	28–85
2 (4464)	82	75–94	74	5	46–92
3 (4331)	105	95–119	78	5	45–106

#### Time

The child's age in months was used as the measure of time. There were approximately 24 months between each wave of the LSAC, with the age of children within each wave varying around a median age (see Table 1). This distribution of ages allowed a detailed month-by-month analysis of growth in receptive vocabulary ability over time.

#### Child, maternal and family candidate predictors

A total of 28 candidate predictor variables were selected for models. These were grouped into child, maternal, family and home environment characteristics (see Table 2).

**Table 2:** Child, maternal and family variables.

*Child variables*	%	*Maternal variables*	%
**Sex**		**Mother's age at birth**	
male	50.9	Teen	2.9
Female^a^	49.1	40+	3.2
		20–39^a^	93.9
**Ethnicity**			
SC ATSI^b^	3.8	**Mother alcohol problem**	
SC non-ATSI^a^	96.2	Yes	12.1
		No^a^	87.9
**Birthweight**			
Low birthweight	6.5	**Mother smoker**	
Normal birthweight^a^	93.5	Yes	23.1
		No^a^	76.9
**Ear infections**			
Yes	7.9	**Mother K6 symptomatic**	
No^a^	92.1	Yes	16.2
		No^a^	83.8
**Who Am I**			
Quintile 1 (lowest)	19.3	**Maternal education**	
Quintile 2	22.4	Year 12	32.3
Quintile 3	16.4	Year 11 or less	39.2
Quintile 4	20.5	University^a^	28.5
Quintile 5 (highest)^a^	21.4		
		**Maternal work hours**	
**Persistence**		Zero hours^c^	43.3
Quintile 1 (lowest)	18.9	Full time: 38 hours+	13.3
Quintile 2	25.8	Part time: 1–37 hours^a^	43.4
Quintile 3	22.7		
Quintile 4	17.4	**Maternal consistency**	
Quintile 5 (highest)^a^	15.2	Quintile 1 (least consistent)	21.7
		Quintile 2	16.1
**Reactivity**		Quintile 3	21.9
Quintile 1 (most reactive)	21.9	Quintile 4	20.4
Quintile 2	17.7	Quintile 5^a^	19.9
Quintile 3	20.4		
Quintile 4	21.0	**Maternal inductive reasoning**	
Quintile 5^a^	19.0	Quartile 1 (lowest reasoning)	16.8
		Quartile 2	33.7
**Sociability**		Quartile 3	22.7
Quintile 1 (lowest)	18.4	Quartile 4^a^	26.8
Quintile 2	24.4		
Quintile 3	21.1	**Maternal warmth**	
Quintile 4	19.2	Quintile 1 (lowest warmth)	21.4
Quintile 5^a^	16.9	Quintile 2	24.4
		Quintile 3	11.9
		Quintile 4	23.2
		Quintile 5^a^	19.1
		**Maternal hostility**	
		Quintile 1 (greatest hostility)	21.6
		Quintile 2	27.8
		Quintile 3	16.4
		Quintile 4	17.7
		Quintile 5^a^	16.5
***Family variables***	%		
**Mother Non-English speaking background**		**Health care card**	
Yes	15.7	Yes	22.0
No^a^	84.3	No^a^	78.0
			
**Family structure**		**Financial hardship**	
Single mother family	14.0	Yes	30.6
Other a	86.0	No^a^	69.4
**Siblings**		**SEIFA disadvantage index**	
One	48.4	Quintile 1 (lowest SEIFA)	21.9
Two	26.7	Quintile 2	20.8
Three	9.3	Quintile 3	19.4
Four or more	4.1	Quintile 4	18.6
Zero^a^	11.5	Quintile 5^a^	19.3
**Family income**		**Reads to child**	
Under $600	17.5	Not at all	3.6
$600–$999	23.9	1–2 days/week	19.5
$1000–$1499	25.0	3–5 days/week	29.7
$1500–$1999	17.0	Daily^a^	47.2
$2000 or more^a^	16.6		
		**Playgroup**	
		No	67.8
		Yes^a^	32.2
		**Hours a week in care**	
		9–20	65.5
		21–30	20.0
		31+	8.2
		8 or less hours^a^	6.3

a Reference category for modelling;

b Aboriginal and Torres Strait Islander;

c Includes not in labour force.

#### Child characteristics

The child characteristics in our models were: Gender, ethnicity, birthweight, ear infections, school readiness and temperament. There were equal proportions of girls and boys in the sample. A small proportion of children (n = 187; 3.8%) were of Aboriginal and/or Torres Strait Islander decent and were coded to distinguish them from those who were not. Primary carers were asked to report their child's birthweight which was subsequently coded into those children who were born with low birthweight (<2500 grams; 6.5%) and those who weighed more than this (> = 2500 grams). Mothers were asked if their child had a range of ongoing health problems. Because of the known association between hearing loss and language emergence a single item indicator of ongoing ear infections at Wave 1 was included. In addition to the Adapted PPVT-III, each study child was directly assessed at Wave 1 using the *Who Am I?* [52]. This is a measure of school readiness and comprises 11 items in which children write their names, copy shapes and write words and numbers. It has been extensively calibrated for use in the LSAC and has well demonstrated item characteristics, high internal reliability (0.89), and excellent distributional properties [52]. In this report, study children have been grouped into quintiles of performance based on the total *Who Am I?* score with high quintiles representing higher levels of performance.

Child temperament was measured at wave 1 of the LSAC by administering the Short Temperament Scale for Children (STSC; [53]) to the person designated as Parent 1. The STSC measures three dimensions of temperament: persistence, reactivity and sociability. Persistence refers to a child's ability to stay focussed on tasks, reactivity refers to refers to irritability, negative mood and high-intensity negative reactions, and sociability refers to a child's tendency to approach novel situations and people or conversely to withdraw and be wary. Each temperament dimension was assessed through parent report using four items, rating the frequency of the behaviours on a 6-point Likert scale of occurrence from “almost never” to “almost always”. Where data were missing for any of the items making up a dimension of temperament respondents were coded as missing for that variable. Four composites were constructed based on the respective items and each was then divided into quintiles with higher quintiles representing the positive aspects of each dimension.

#### Maternal characteristics

The maternal characteristics in our models were: Age at the birth of the child, problematic alcohol use, smoking, mental health distress, education, hours of paid employment and parenting. The biological mother's age at the birth of the child was grouped into categories representing teen birth (<age 20 years), 20–39 years and 40 or more years at birth with the vast majority of mothers (93.9%) of study children in the age range 20–39 years.

Information on current tobacco and alcohol at Wave 1 was gathered from the mothers. We defined problematic alcohol use where women reported their daily alcohol consumption to exceed 2 standard drinks and/or where they reported frequent binge drinking of 5 or more alcoholic drinks at least 2–3 times per month with 12.1% being so classified. Study children's mothers were asked about tobacco use and also categorised as either current smokers (23.1%) or not current smokers.

In this study, we used the Kessler-6 (K-6) to measure maternal non-specific psychological distress. Women with scores of 8 or more were classified as having symptomatic psychological distress. This threshold is consistent with other studies [54], [55] using the K-6. The scale has robust characteristics as an indicator of mental health with recent Australian findings [39] that 53% of Australian adults with a score of 8–12 on the K6 had an ICD-10 anxiety or depressive disorder as diagnosed by the Composite International Diagnostic Interview. Sixteen percent of mothers reported symptomatic psychological distress.

In Australia, at the time of this study, 10 years of education was compulsorily mandated. Maternal education in years was grouped into three levels according to those who had completed 11 years (39.2%), 12 years (32.3%), and those who had completed more than 12 years (i.e. University education) (28.5%).

Mothers were variously employed at the time when the children were first measured. We used total hours of paid maternal employment to distinguish mothers who were not in paid employment (0 hours), from those in part time paid employment (1–37 hours; Mean = 17.8) and in full time paid employment (> = 38 hours; Mean = 44.9). Equal proportions of women (43%) were either not in paid employment or working part time with the remainder (13%) working full time.

The parenting characteristics of both parents were measured in a self-complete form, using four measures of parenting warmth, hostility, consistency and inductive reasoning developed for the LSAC [54]. We use the mother's responses in this report. Responses to each item were on a 5-point Likert scale, ranging from “almost never” to “always/almost always”. Items for each measure were summed to create a composite score with higher levels representing more positive parenting characteristics. Item and scale properties for the LSAC parenting measures have been extensively documented [54]. Ordinal scale reliabilities [55] were 0.72 for maternal hostility, 0.82 for consistency and .93 and .94 for warmth and inductive reasoning respectively.

#### Family characteristics

The characteristics of the family home environment in our models were: NESB, family structure, sibship size, income, health care card, financial hardship, socioeconomic disadvantage, reading to the study child, playgroup and child care. As the focus of this study is explicitly on English language development and because language development is known to vary where more than one language is spoken in the home, we used the mother's NESB as a general indicator for language other than English spoken in the household at Wave 1. About 16% of mothers were predominately non-English speaking at the time of the interview.

With respect to family composition, two variables were selected as candidate predictors of vocabulary development: Family structure (sole parent vs. other) and number of siblings (0, 1, 2, 3, 4+). About 13% of the study children were living in single mother families and the majority had one sibling (48.4%) or were singletons (11.4%) at the time of the Wave 1 interview. The study design did not permit the establishment of birth order.

Families were asked to report their total weekly family income from all sources. Responses were partitioned into relatively equal quintiles: those families earning under $600, $600–$999, $1000–$1499, $1500–$1999, and $2000 or more per week. In Australia, there is universal health coverage and where income falls below a defined threshold and/or certain hardship criteria are met families also qualify for a health care card. About 22.0% of LSAC families had a health care card and this is used as an indicator of financial need in the LSAC families. Additionally, an indicator of family hardship was also derived where families reported, due to shortage of money over the last 12 months that: they had not been able to pay gas, electricity or telephone bills on time; they had not been able to pay the mortgage or rent on time; adults or children had gone without meals; they family had been unable to heat or cool their home; they had pawned or sold something; or sought assistance from a welfare or community organisation. About one third of families reported at least one of these occurrences in the previous 12 months to the Wave 1 interview.

An area measure of socioeconomic disadvantage was also estimated for each participating family. The family home was coded with Socio-Economic Indicators for Area (SEIFA) disadvantage, indexed in quintiles–higher quintiles represent greater levels of disadvantage. The neighbourhood SEIFA disadvantage index summarizes information from the Australian Census of Population and Housing as this relates to economic and social disadvantage in small areas, such as low income, low educational attainment and high unemployment [56]. This data was linked at either the Statistical Local Area (SLA) level or, where this was not available, the child's postcode.

Several indicators of the child's learning environment were gathered. The frequency with which the primary caregiver read to the study child was assessed via face-to-face interview. A total of 182 (3.6%) of parents reported not reading to the child at all, 970 (19.5%) reported reading 1 or 2 days a week, 1478 (29.7%) reported reading to the child 3–5 days a week, and 2350 (47.2%) reported reading to the child daily. Mothers were asked if their child had attended a playgroup in the period 12 months prior to the Wave 1 interview with about one third indicating this to be the case. Finally, hours a week in care/early education were coded by asking the parent “how many hours a week on average does the child go to school, kindergarten, pre-school, and/or day-care?” A total of 298 (6.3%) attended 8 or less hours a week, 3114 (65.5%) attended 9–20 hours a week, 950 (20.0%) attended 21–30 hours a week, and 389 (8.2%) attended 31+ hours a week.

### Data Analysis

The data were analysed using growth curve models [57]providing a clearer view of the trajectories of vocabulary growth. These models estimate intercept and slope effects of receptive vocabulary development in relation to the candidate predictors. Predictors may be examined in both bivariate and multivariate models. In multivariate models the predictor set is simultaneously adjusted permitting an estimate of the relative (e.g. unique) contribution of each of the predictors when the remaining predictors are at their reference levels. A desirable feature of growth curve analysis is its use of unevenly spaced measurements and its use of all available data in the presence of some level of missing data [53].

Our growth curve modelling utilises a two-level nested structure. Level 1 is the within-person model while level 2 is the between-person model. The within-person component allows each child to have a unique receptive vocabulary growth trajectory and the between-person component of the models represents variation in receptive vocabulary growth parameters among children with similar characteristics. The Proc Mixed procedure in SAS 9.2 was used to fit these models. As we are primarily interested in the fixed-effects portions of these models, we have used Maximum Likelihood as the estimation method.

Our analyses proceeded in three steps.

First, an unconditional growth model was estimated to gain an overall model of receptive vocabulary development across the sample. The only predictor variable in this unconditional growth model was time (i.e., age). We made the decision to input time to the model parameterized as the child's age minus 50 months. Thus each child's age was modelled as his or her age at each wave minus 50 (i.e. age at wave 1–50, age at wave 2–50, age at wave 3–50). This set the intercept (i.e. the start of the growth trajectory) such that the youngest child in the study had an intercept of zero months at Wave 1.

Receptive vocabulary development was then modelled as a linear function of children's initial receptive vocabulary (intercept), growth per month in receptive vocabulary (linear slope), and random error. Following Singer and Willett [57], the unconditional growth model is represented by the following equation:

PPVT_ij_  =  π_oj_ + π_1i_ (AGE_ij_ − 50) + ε_ii_

where

π_oj_  =  γ_00_ + ζ_0i_

π_1j_  =  γ_10_ + ζ_1i_

That is, PPVT_ij_  =  (γ_00_ + ζ_0i_) + (γ_10_ + ζ_1i_) (AGE_ij_ − 50) + ε_ii_

Or alternatively,

PPVT_ij_  =  γ_00_ + γ_10_ (AGE_ij_ − 50) + ζ_0i_ + ζ_1i_) (AGE_ij_ − 50) + ε_ii_

In this model PPVT_ij_ represents child i's PPVT score at time j, π_oj_ represents child i's PPVT score at an initial intercept (time zero), π_1i_ represents a linear function of PPVT score at child's age in months, AGE_ij_ represents child i's age at each time j and ε_ii_ represents unique error associated with that child at time j. γ_00_ and γ_10_ represent systematic between-person differences in intercept and slope. ζ_0i_ and ζ_1i_ represent the residuals unexplained by the between-person parameters. These residuals represent the amount of variation left over after accounting for the model's predictors, in this case, the child's age.

**Figure 1:**
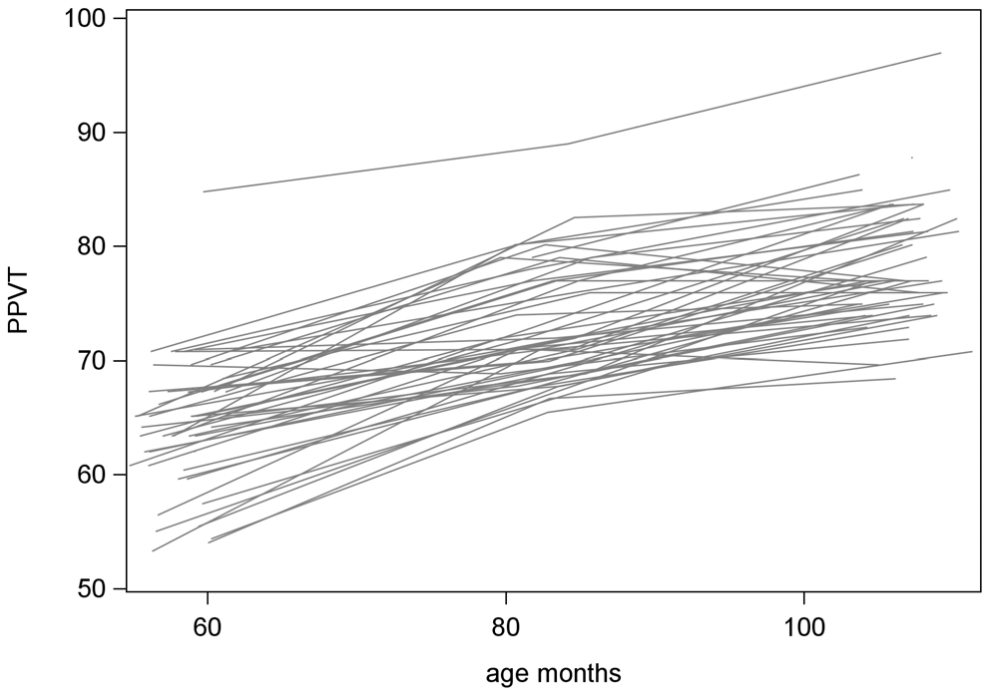
Empirical growth plots (n = 50).

In this model we presume that a linear trajectory adequately describes growth over time in this study. This assumption was supported by an extensive visual screening of initial growth plots. Figure 1 depicts the empirical growth plots for a random sample of 50 children. There were no major departures from linearity, and with a maximum of three time points per child, a linear model was determined to be adequate to describe each child's trajectory.

Second, we developed a series of conditional models that included child, maternal and family predictors. Initially, each predictor was considered individually. Each sub-model fitted into the above model as follows:

π_oj_  =  γ_00_ + γ_01_PREDICTOR + ζ_0i_

π_1j_  =  γ_10_ + γ_11_PREDICTOR + ζ_1i_

In these models the terms γ_00_, γ_01_, γ_10_, and γ_11_, represent fixed effects. These are the systematic between-individual differences in intercepts and slopes according to the predictors in the model. γ_01_ represented the difference between the reference group and the group of interest at intercept and γ_11_ represented the difference between the reference group and the group of interest in slope. As before, ζ_0i_ and ζ_1i_ represented the residuals that were not explained by the predictors.

Our fixed effects were typically parameterized such that the lowest risk (or most advantaged) group represented our reference group. For each variable, the percentage of the sample in each of the reference categories, along with the percentage of the sample in each of the other categories are shown in Table 2. For example, in examining birthweight we used children of ‘normal’ birthweight as our reference group. For school readiness, we used children in the quintile with the highest *Who Am I?* scores as our reference group. Thus, we estimated the bivariate impact on the child's PPVT score at intercept (i.e. at the start of the growth trajectory) and slope (i.e., monthly rate of growth in PPVT score) for each candidate predictor. To establish the effect size for the various predictors we standardised the regression coefficients from the growth curve models into standard deviation units (Cohen's *d*) cohen [58].

Following Singer and Willett [57] we calculated a pseudo-R^2^ measure of model fit by squaring the correlation between observed and predicted values for PPVT, as an indicator of model fit.

Third and finally, we tested an overall multivariate model by selecting candidate predictors from these bivariate estimates on the basis of their Cohen's *d*. Cohen provides some general guidance on what constitutes a negligible (*d*<*0.2*), small (*d* = 0.2), medium (*d* = 0.5) and large (*d* = 0.8) effect sizes. We were able to calculate effect sizes across a range of values of change in the PPVT score and chose *d* = 0.3 as the threshold for including a predictor in the multivariate model. We chose this threshold because an effect size of this magnitude corresponds to about 6 months of development with respect to change in the PPVT score. Our rationale was that a six-month lag in receptive vocabulary ability at 42 months constitutes a developmentally meaningful gap. It was of interest to determine the extent to which children with risk factors associated with a 6 month lag in receptive vocabulary abilities at 4 years were able to close the gap at 8 years. We acknowledge that there is no precedent for setting a threshold of *d* = 0.3 as a developmentally meaningful effect size in relation to language ability. However, we were guided by a meta-analysis of meta-analyses of child and family influences on children's educational achievement that identified *d* = 0.31 as the average effect size for child influences and *d* = 0.40 for family influences [59]. Our rationale was not to reduce the predictor set a priori, but to select predictors for multivariate modelling associated with a developmentally meaningful lag in receptive vocabulary ability at the intercept. Using the threshold of *d* = 0.3, a subset of 16 predictors from the 28 were selected for entry into multivariate modelling, allowing estimation of their independent effects on the PPVT score intercept and slope. We examined model diagnostics and noted that after accounting for the predictors in the multivariate model, random slope (i.e. individual rate of growth in PPVT scores) no longer accounted for any substantive variation in PPVT score, to the point that there was not enough slope variance to model. As a result, random slope was removed from the final model. So the final model included a random intercept term, and co-efficients for different rates of growth for each level of each predictor variable.

## Results

Growth modelling permits several views of vocabulary change over time.

Table 3 contains the estimates of the unconditional growth model and the bivariate growth model along with the effects for each predictor on the initial PPVT score (the intercept) and the effect of the predictor on PPVT growth (the slope). These effects include the predictor's effect on the PPVT score at intercept, its effect size – Cohen's d (*d*_i_), and the number of months this effect represents relative to the reference group for the given predictor variable. Additionally we provide estimates of each predictor's effect on growth in the PPVT score over time (i.e., slope), the effect size for the slope calculated as Cohen's *d* (*d*_s_), and then the projected change in PPVT from 50 months onward to 57, 82 and 105 months (the median ages at each wave of the study). The final columns in this table provide the estimated difference in PPVT points and associated difference in developmental age in months, along with model fit indices.

**Table 3:** Bivariate associations between child, maternal and family characteristics and receptive vocabulary growth 4–8 years.a,b

Variables	Initial effect			Growth rate		PPVT scores at 50, 57, 82 and 105 months				Difference @105 months
	**Intercept**	**Cohen's ** ***d*** ** (** ***d*** **_i_)**	**Months**	**Growth Differential/** ***Slope***	**Cohen's ** ***d*** ** (** ***d*** **_s_)**	**50**	**57**	**82**	**105**	**PPVT points/Months**
**Unconditional growth model**	62.8838 (62.6978, 63.0697)			0.29 (0.2862, 0.2937)		62.88	64.91	72.16	78.83	
***Child variables***										
**Sex**										
male	−0.7392 (−1.1105, −0.368)	−0.12 (−0.18, −0.06)	−2.45	0.02391 (0.01651, 0.03131)	0.19 (0.13, 0.25)	62.52	64.63	72.17	79.11	0.58 (2.07)
*female (ref.)*	*63.2583 (62.9939, 63.5227)*			*0.2778 (0.2725, 0.2831)*		*63.26*	*65.20*	*72.15*	*78.54*	
**Ethnicity**										
SC ATSI	−2.5233 (−3.5538, −1.4928)	−0.4 (−0.57, −0.24)	−8.53	0.005966 (−0.0158, 0.02774) (n.s.)	0.05 (−0.13,0.22)	60.45	62.52	69.91	76.71	−2.20 (−7.42)
*SC non-ATSI (ref.)*	*62.9719 (62.7832, 63.1606)*			*0.2897 (0.2859, 0.2935)*		*62.97*	*65.00*	*72.24*	*78.91*	
**Birthweight**										
Low birthweight	−2.1437 (−2.9145, −1.373)	−0.34 (−0.47, −0.22)	−7.00	0.01745 (0.00198, 0.03292)	0.14 (0.02, 0.26)	60.92	63.06	70.71	77.75	−1.18 (−3.87)
*Normal birthweight (ref.)*	*63.0604 (62.8684, 63.2525)*			*0.2887 (0.2849, 0.2926)*		*63.06*	*65.08*	*72.30*	*78.94*	
**Ear infections**										
Yes	−0.6953 (−1.3893,−0.00125)	−0.11 (−0.22, 0)	−2.35	0.006838 (−0.00708, 0.02076)	0.05 (−0.06,0.17)	62.24	64.32	71.72	78.54	−0.32 (−1.08)
*No (ref.)*	*62.9379 (62.7444, 63.1315)*			*0.2894 (0.2856, 0.2933)*		*62.94*	*64.96*	*72.20*	*78.85*	
***Who Am I?***										
quintile 1 (lowest)	−4.5487 (−5.1206, −3.9769)	−0.73 (−0.82, −0.64)	−14.52	0.03721 (0.02549, 0.04893)	0.30 (0.2, 0.39)	60.30	62.50	70.33	77.54	−2.50 (−7.99)
quintile 2	−2.7838 (−3.3288, −2.2387)	−0.45 (−0.53, −0.36)	−9.30	0.0231 (0.01205, 0.03416)	0.19 (0.1, 0.27)	62.07	64.16	71.64	78.52	−1.51 (−5.06)
quintile 3	−1.478 (−2.0707, −0.8853)	−0.24 (−0.33, −0.14)	−5.22	0.007213 (−0.00482, 0.01925) (n.s.)	0.06 (−0.04,0.15)	63.37	65.36	72.44	78.96	−1.08 (−3.82)
quintile 4	−0.9155 (−1.4749, −0.356)	−0.15 (−0.24, −0.06)	−3.30	0.001094 (−0.01023, 0.01242) (n.s.)	0.01 (−0.08, 0.1)	63.94	65.88	72.81	79.18	−0.86 (−3.09)
*quintile 5 (ref.)*	*64.8521 (64.4592, 65.2449)*			*0.2761 (0.2682, 0.284)*		*64.85*	*66.78*	*73.69*	*80.04*	
***Persistence***										
Quintile 1 (lowest persistence)	−2.5931 (−3.2727,−1.9135)	−0.42 (−0.52,−0.31)	−8.78	0.0135 (−0.0002, 0.02719) (n.s.)	0.11 (0, 0.22)	61.70	63.76	71.15	77.95	−1.85 (−6.26
quintile 2	−1.5446 (−2.1788,−0.9104)	−0.25 (−0.35,−0.15)	−5.27	0.01127 (−0.00143, 0.02397) (n.s.)	0.09 (−0.01,0.19)	62.74	64.80	72.13	78.87	−0.92 (−3.15)
quintile 3	−0.5575 (−1.2062, 0.09123) (n.s.)	−0.09 (−0.19, 0.01)	−1.97	0.000333 (−0.01266, 0.01333) (n.s.)	0 (−0.1, 0.11)	63.73	65.71	72.77	79.26	−0.54 (−1.91)
quintile 4	−0.3652 (−1.0521, 0.3217) (n.s.)	−0.06 (−0.17, 0.05)	−1.31	−0.00424 (−0.01801, 0.00952) (n.s.)	−0.03 (−0.14,0.08)	63.92	65.87	72.81	79.20	−0.60 −2.15
*quintile 5 (ref.)*	*64.2885 (63.786, 64.7909)*			*0.282 (0.272, 0.2921)*		*64.29*	*66.26*	*73.31*	*79.80*	
**Reactivity**										
quintile 1 (most reactive)	−2.5405 (−3.1644, −1.9166)	−0.41 (−0.51, −0.31)	−8.50	0.02073 (0.008149,0.03331)	0.17 (0.07, 0.27)	61.74	63.83	71.30	78.18	−1.40 (−4.68)
quintile 2	−1.0236 (−1.6746, −0.3726)	−0.16 (−0.27, −0.06)	−3.60	0.005785 (−0.00735, 0.01892) (n.s.)	0.05 (−0.06,0.15)	63.25	65.24	72.34	78.87	−0.71 (−2.48)
quintile 3	−0.9774 (−1.6047,−0.3501)	−0.16 (−0.26,−0.06)	−3.39	0.01028 (−0.00233, 0.02289) (n.s.)	0.08 (−0.02,0.18)	63.30	65.32	72.53	79.17	−0.41 (−1.43)
quintile 4	−0.5461 (−1.1683, 0.07606) (n.s.)	−0.09 (−0.19, 0.01)	−1.94	0.003802 (−0.00871,0.01631) (n.s.)	0.03 (−0.07,0.13)	63.73	65.71	72.76	79.24	−0.34 (−1.19)
*quintile 5 (ref.)*	*64.2773 (63.8255, 64.7291)*			*0.2782 (0.2691, 0.2873)*		*64.28*	*66.22*	*73.18*	*79.58*	
**Sociability**										
quintile 1 (lowest sociability)	−1.3928 (−2.0597,−0.7258)	−0.22 (−0.33,−0.12)	−4.85	0.004047 (−0.00927, 0.01737) (n.s.)	0.03 (−0.07,0.14)	62.50	64.51	71.69	78.30	−1.17 (−4.07)
quintile 2	−0.9879 (−1.6177,−0.3581)	−0.16 (−0.26,−0.06)	−3.40	0.007086 (−0.00552, 0.01969) (n.s.)	0.06 (−0.04,0.16)	62.90	64.93	72.19	78.87	−0.60 (−2.06)
quintile 3	−0.6205 (−1.2692, 0.02808) (n.s.)	−0.1 (−0.2, 0)	−2.18	0.00157 (−0.01143, 0.01457) (n.s.)	0.01 (−0.09,0.12)	63.27	65.26	72.38	78.94	−0.53 (−1.88)
quintile 4	−0.3166 (−0.9761, 0.3429) (n.s.)	−0.05 (−0.16, 0.05)	−1.10	0.005024 (−0.00812, 0.01817) (n.s.)	0.04 (−0.07,0.15)	63.57	65.59	72.80	79.43	−0.04 (−0.14)
*quintile 5 (ref.)*	*63.8893 (63.4059, 64.3726)*			*0.2833 (0.2736, 0.2929)*		*63.89*	*65.87*	*72.95*	*79.47*	
***Maternal variables***										
**Mothers Age at birth**										
Teen	−1.9679 (−3.133,−0.8028)	−0.32 (−0.5,−0.13)	−6.67	0.005719 (−0.01874, 0.03018) (n.s.)	0.05 (−0.15,0.24)	60.97	63.04	70.41	77.20	−1.65 (−5.60)
40+	0.8017 (−0.2589, 1.8622) (n.s.)	0.13 (−0.04, 0.3)	2.67	0.01096 (−0.01054, 0.03245) (n.s.)	0.09 (−0.08,0.26)	63.74	65.84	73.35	80.26	1.40 (4.68)
*20*−*39 (ref.)*	*62.9374 (62.7459, 63.129)*			*0.2894 (0.2855, 0.2932)*		*62.94*	*64.96*	*72.20*	*78.85*	
**Mother alcohol problem**										
Yes	−0.5181 (−1.1332, 0.09709) (n.s.)	−0.08 (−0.18, 0.02)	−1.81	−0.00185 (−0.0142, 0.01049) (n.s.)	−0.01 (−0.11,0.08)	62.78	64.78	71.93	78.50	−0.62 (−2.17)
*No (ref.)*	*63.3021 (63.0884, 63.5157)*			*0.2876 (0.2833, 0.2919)*		*63.30*	*65.32*	*72.51*	*79.12*	
**Mother smoker**										
Yes	−1.5548 (−2.0238,−1.0857)	−0.25 (−0.32,−0.17)	−5.34	0.005464 (−0.00404, 0.01497) (n.s.)	0.04 (−0.03,0.12)	62.04	64.07	71.35	78.04	−1.25 (−4.31)
*No (ref.)*	*63.5912 (63.3676, 63.8147)*			*0.2855 (0.281, 0.29)*		*63.59*	*65.59*	*72.73*	*79.29*	
Mother K6 symptomatic										
Yes	−2.6239 (−3.1603,−2.0874)	−0.42 (−0.51,−0.33)	−8.41	0.02991 (0.01902, 0.0408)	0.24 (0.15, 0.33)	61.03	63.21	71.01	78.19	−0.98 (−3.14)
										
*No (ref.)*	*63.652 (63.4386, 63.8655)*			*0.2821 (0.2778, 0.2864)*		*63.65*	*65.63*	*72.68*	*79.17*	
										
**Maternal education**										
Year 12	−1.8697 (−2.334,−1.4053)	−0.3 (−0.37,−0.23)	−6.46	0.003016 (−0.00637, 0.0124) (n.s.)	0.02 (−0.05, 0.1)	62.81	64.84	72.08	78.73	−1.70 (−5.89)
Year 11 or less	−3.004 (−3.4531,−2.5549)	−0.48 (−0.55,−0.41)	−10.31	0.004849 (−0.00429, 0.01398) (n.s.)	0.04 (−0.03,0.11)	61.68	63.72	71.00	77.70	−2.74 (−9.40)
*University (ref.)*	*64.6844 (64.346, 65.0228)*			*0.2864 (0.2796, 0.2932)*		*64.68*	*66.69*	*73.85*	*80.44*	
**Maternal work hours**										
zero hours (includes not in labour force.)	−2.0799 (−2.4751,−1.6846)	−0.33 (−0.4,−0.27)	−6.96	0.01877 (0.0108, 0.02674)	0.15 (0.09,0.21)	61.80	63.89	71.36	78.23	−1.05 (−3.51)
full-time: 38 hours +	−0.7668 (−1.3417,−0.1919)	−0.12 (−0.21,−0.03)	−2.62	0.01319 (0.001648,0.02473)	0.11 (0.01, 0.2)	63.11	65.16	72.49	79.24	−0.04 (−0.14)
*part-time: 1-37* *hours (ref)*	*63.878 (63.601, 64.156)*			*0.2800* (*0.2745, 0.2856)*		63.88	*65.84*	*72.84*	*79.28*	
**Maternal consistency**										
quintile 1 (least consistency)	−3.4574 (−4.0245,−2.8903)	−0.55 (−0.64,−0.46)	−11.39	0.02709 (0.01549, 0.03868)	0.22 (0.12, 0.31)	60.79	62.91	70.50	77.48	−1.97 (−6.48)
quintile 2	−2.0672 (−2.6727,−1.4617)	−0.33 (−0.43,−0.23)	−7.02	0.01802 (0.005691,0.03036)	0.14 (0.05, 0.24)	62.18	64.24	71.60	78.37	−1.08 (−3.65)
quintile 3	−1.0525 (−1.6135,−0.4916)	−0.17 (−0.26,−0.08)	−3.58	0.01757 (0.006188,0.02895)	0.14 (0.05, 0.23)	63.20	65.25	72.60	79.36	−0.09 (−0.29)
quintile 4	−0.1647 (−0.7314, 0.402) (n.s.)	−0.03 (−0.12, 0.06)	−0.59	0.00467 (−0.0068, 0.01613) (n.s.)	0.04 (−0.05,0.13)	64.08	66.05	73.08	79.54	0.09 (0.33)
*quintile 5 (ref.)*	*64.2476 (63.846, 64.6492)*			*0.2764 (0.2682, 0.2845)*		*64.25*	*66.18*	*73.09*	*79.45*	
**Maternal inductive reasoning**										
quartile 1 (lowest reasoning)	−1.2547 (−1.837,−0.6723)	−0.2 (−0.29,−0.11)	−4.25	0.006295 (−0.00549, 0.01808) (n.s.)	0.05 (−0.04,0.14)	61.89	63.95	71.34	78.13	−0.91 (−3.08)
quartile 2	0.02765 (−0.4515, 0.5068) (n.s.)	0 (−0.07, 0.08)	0.10	0.000543 (−0.00911, 0.01019) (n.s.)	0 (−0.07,0.08)	63.17	65.20	72.44	79.10	0.06 (0.20)
quartile 3	−0.1014 (−0.6308, 0.4279) (n.s.)	−0.02 (−0.1, 0.07)	−0.35	−0.00203 (−0.01267, .008603) (n.s.)	−0.02 (−0.1, 0.07)	63.04	65.05	72.22	78.82	−0.21 (−0.74)
*quartile 4 (ref.)*	*63.1425 (62.784, 63.501)*			*0.289 (0.2818, 0.2963)*		*63.14*	*65.17*	*72.39*	*79.04*	
**Maternal warmth**										
quintile 1 (lowest warmth)	−0.3784 (−0.9648, 0.208) (n.s.)	−0.06 (−0.15, 0.03)	−1.27	0.01629 (0.004489, 0.0281)	0.13 (0.04, 0.23)	62.59	64.67	72.10	78.94	0.52 (1.74)
quintile 2	0.1713 (−0.3977, 0.7403) (n.s.)	0.03 (−0.06, 0.12)	0.59	0.01166 (0.000201,0.02312)	0.09 (0, 0.19)	63.14	65.19	72.50	79.23	0.81 (2.78)
quintile 3	0.1806 (−0.5121, 0.8732) (n.s.)	0.03 (−0.08, 0.14)	0.64	0.00167 (−0.01222, 0.01557) (n.s.)	0.01 (−0.1, 0.12)	63.15	65.13	72.19	78.69	0.27 (0.96)
quintile 4	−0.1323 (−0.7075, 0.4428) (n.s.)	−0.02 (−0.11, 0.07)	−0.46	0.009639 (−0.00194, 0.02122) (n.s.)	0.08 (−0.02,0.17)	62.84	64.87	72.14	78.82	0.40 (1.37)
*quintile 5 (ref.)*	*62.971 (62.5435, 63.3986)*			*0.2809 (0.2722, 0.2895)*		*62.97*	*64.94*	*71.96*	*78.42*	
**Maternal hostility**										
quintile 1 (greatest hostility)	−1.1903 (−1.7982,−0.5825)	−0.19 (−0.29,−0.09)	−4.02	0.005552 (−0.00668, 0.01778) (n.s.)	0.04 (−0.05,0.14)	62.07	64.14	71.55	78.36	−0.88 (−2.99)
quintile 2	−0.4565 (−1.0313, 0.1183) (n.s.)	−0.07 (−0.17, 0.02)	−1.56	0.001777 (−0.00978, 0.01334) (n.s.)	0.01 (−0.08,0.11)	62.80	64.85	72.16	78.89	−0.36 (−1.23)
quintile 3	−0.1448 (−0.7907, 0.5012) (n.s.)	−0.02 (−0.13, 0.08)	−0.51	−0.00705 (−0.02001,0.00590) (n.s.)	−0.06 (−0.16 0.05)	63.11	65.10	72.19	78.71	−0.53 (−1.88)
quintile 4	0.3726 (−0.2579, 1.0031) (n.s.)	0.06 (−0.04, 0.16)	1.32	−0.00762 (−0.02029,0.00505) (n.s.)	−0.06 (−0.16,0.04)	63.63	65.61	72.69	79.20	−0.05 (−0.16)
*quintile 5 (ref.)*	*63.2569 (62.8023, 63.7114)*			*0.2907 (0.2816, 0.2998)*		*63.26*	*65.29*	*72.56*	*79.25*	
***Family variables***										
**Mother Non-English Speaking Background**										
Yes	−4.5928 (−5.1001,−4.0856)	−0.74 (−0.82,−0.65)	−16.34	0.06062 (0.05007, 0.07117)	0.49 (0.4, 0.57)	63.58	65.55	72.58	79.04	−1.26 (−4.48)
*No (ref.)*	*68.1767 (67.5679, 68.7854)*			*0.2204 (0.2079, 0.233)*		*68.18*	*69.72*	*75.23*	*80.30*	
Family Structure										
Single mother family	−1.8022 (−2.3468,−1.2577)	−0.29 (−0.38,−0.2)	−6.01	0.01169 (0.000501,0.02287)	0.09 (0,0.18)	61.33	63.43	70.93	77.83	−1.16 (−3.86)
*Other (ref.)*	*63.128 (62.9293, 63.3268)*			*0.2883 (0.2843, 0.2923)*		*63.13*	*65.15*	*72.35*	*78.98*	
**Family Structure**										
Single mother family	−1.8022 (−2.3468,−1.2577)	−0.29 (−0.38,−0.2)	−6.01	0.01169 (0.000501,0.02287)	0.09 (0,0.18)	61.33	63.43	70.93	77.83	−1.16 (−3.86)
*Other (ref.)*	*63.128 (62.9293, 63.3268)*			*0.2883 (0.2843, 0.2923)*		*63.13*	*65.15*	*72.35*	*78.98*	
**Siblings**										
One	0.1972 (−0.4127, 0.8072) (n.s.)	0.03 (−0.07, 0.13)	0.69	−0.00939 (−0.02181,0.00303)	−0.08 (−0.17,0.02)	63.58	65.57	72.69	79.24	−0.32 (−1.12)
Two	−0.8291 (−1.4838,−0.1745)	−0.13 (−0.24,−0.03)	−2.86	−0.00441 (−0.01771,0.00889) (n.s.)	−0.04 (−0.14,0.07)	62.55	64.58	71.82	78.48	−1.07 (−3.70)
Three	−2.3054 (−3.123,−1.4877)	−0.37 (−0.5,−0.24)	−7.58	0.009904 (−0.00682,0.02663) (n.s.)	0.08 (−0.05,0.21)	61.08	63.20	70.80	77.80	−1.76 (−5.79)
four plus	−3.9384 (−5.0231,−2.8538)	−0.63 (−0.8,−0.46)	−12.69	0.01626 (−0.00633, 0.03885) (n.s.)	0.13 (−0.05,0.31)	59.44	61.61	69.37	76.51	−3.04 (−9.81)
*zero (ref.)*	*63.3804 (62.8299, 63.9309)*			*0.2941 (0.2829, 0.3054)*		*63.38*	*65.44*	*72.79*	*79.56*	
**Family income per week**										
Under $600	−4.3259 (−4.9753, −3.6766)	−0.69 (−0.8,−0.59)	−13.90	0.03037 (0.01695, 0.04379)	0.24 (0.14, 0.35)	60.61	62.79	70.57	77.73	−2.66 (−8.53)
$600–$999	−2.9639 (−3.5613, −2.3664)	−0.47 (−0.57,−0.38)	−10.21	0.009318 (−0.00285, 0.02148) (n.s.)	0.07 (−0.02,0.17)	61.97	64.01	71.26	77.94	−2.45 (−8.45)
$1000–$1499	−1.6037 (−2.1903,−1.0172)	−0.26 (−0.35,−0.16)	−5.63	0.00394 (−0.00793, 0.01581) (n.s.)	0.03 (−0.06,0.13)	63.33	65.33	72.45	79.00	−1.39 (−4.87)
$1500–$1999	−1.0349 (−1.6761,−0.3936)	−0.17 (−0.27,−0.06)	−3.63	0.004153 (−0.00882, 0.01713) (n.s.)	0.03 (−0.07 0.14)	63.90	65.90	73.02	79.58	−0.81 (−2.83)
*$2000 or more (ref.)*	*64.9376 (64.4809, 65.3942)*			*0.2809 (0.2716, 0.2901)*		*64.94*	*66.90*	*73.93*	*80.39*	
**Health care card**										
Yes	−2.235 (−2.6882, −1.7819)	−0.36 (−0.43, −0.29)	−7.52	0.009475 (0.000188,0.01876)	0.08 (0, 0.15)	61.13	63.21	70.64	77.48	−1.71 (−5.77)
*No (ref.)*	*63.36 (63.1531, 63.567)*			*0.2878 (0.2836, 0.2919)*		*63.36*	*65.37*	*72.57*	*79.19*	
**Financial hardship**										
Yes	−1.7858 (−2.1897, −1.3818)	−0.29 (−0.35, −0.22)	−5.98	0.01218 (0.003982,0.02037)	0.1 (0.03, 0.16)	61.64	63.73	71.19	78.05	−1.12 (−3.74)
*No (ref.)*	*63.4254 (63.2051, 63.6456)*			*0.2862 (0.2818, 0.2906)*		*63.43*	*65.43*	*72.58*	*79.17*	
**SEIFA disadvantage index**										
quintile 1 (lowest SEIFA)	−1.9619 (−2.5444,−1.3795)	−0.31 (−0.41,−0.22)	−6.81	−0.01421 (−0.02591, −0.0025)	−0.11 (−0.21,−0.02)	61.71	63.73	70.93	77.56	−2.74 (−9.52)
quintile 2	−0.7531 (−1.3375,−0.1687)	−0.12 (−0.21,−0.03)	−2.70	−0.02377 (−0.03549,−0.01205)	−0.19 (−0.28,−0.1)	62.92	64.87	71.83	78.24	−2.06 (−7.39)
quintile 3	−1.0408 (−1.6357,−0.4458)	−0.17 (−0.26,−0.07)	−3.52	−0.00641 (−0.01834,0.00552) (n.s.)	−0.05 (−0.15,0.04)	62.63	64.70	72.10	78.91	−1.39 (−4.71)
quintile 4	−0.01438 (−0.6161, 0.5873) (n.s.)	0 (−0.1, 0.09)	−0.05	−0.01721 (−0.02924,−0.00519)	−0.14 (−0.23,−0.04)	63.66	65.65	72.78	79.34	−0.96 (−3.37)
*quintile 5 (ref.)*	*63.6712 (63.2468, 64.0955)*			*0.3024 (0.2939,0.3109)*		*63.67*	*65.79*	*73.35*	*80.30*	
**Reads to child**										
Not at all	−6.1416 (−7.1978,−5.0853)	−0.98 (−1.15,−0.81)	−18.57	0.04764 (0.02557,0.06971)	0.38 (0.2, 0.56)	58.08	60.39	68.66	76.26	−3.52 (−10.65)
1–2 days/week	−3.3813 (−3.8689,−2.8937)	−0.54 (−0.62,−0.46)	−11.17	0.01966 (0.00965,0.02968)	0.16 (0.08, 0.24)	60.84	62.95	70.52	77.48	−2.30 (−7.60)
3–5 days/week	−1.6242 (−2.046,−1.2024)	−0.26 (−0.33,−0.19)	−5.63	0.005515 (−0.00312, 0.01415) (n.s.)	0.04 (−0.03, 0.11)	62.59	64.61	71.83	78.46	−1.32 (−4.58)
*Daily (ref.)*	*64.2172 (63.9569, 64.4774)*			*0.283 (0.2777,0.2883)*		*64.22*	*66.20*	*73.27*	*79.78*	
**Playgroup**										
No	−0.6133 (−1.0355,−0.1912)	−0.1 (−0.17,−0.03)	−2.13	0.002117 (−0.00629, 0.01053) (n.s.)	0.02 (−0.05, 0.08)	63.00	65.02	72.21	78.83	−0.50 (−1.73)
*Yes (ref.)*	*63.6161 (63.2694, 63.9627)*			*0.2856 (0.2787, 0.2925)*		63.62	65.62	72.76	79.32	
**Hours/week in care**										
9–20	0.3302 (−0.4509, 1.1114) (n.s.)	0.05 (−0.07, 0.18)	1.15	0.003002 (−0.01293, 0.01894) (n.s.)	0.02 (−0.1, 0.15)	63.16	65.17	72.37	78.98	0.50 (1.72)
21–30	0.0689 (−0.7901, 0.9279) (n.s.)	0.01 (−0.13, 0.15)	0.24	0.002743 (−0.01468, 0.02017) (n.s.)	0.02 (−0.12, 0.16)	62.89	64.91	72.10	78.71	0.22 (0.76)
31+	−0.6374 (−1.6354, 0.3607) (n.s.)	−0.1 (−0.26, 0.06)	−2.14	0.01374 (−0.00642, 0.0339) (n.s.)	0.11 (−0.05, 0.27)	62.19	64.28	71.74	78.61	0.12 (0.40)
*8 or less hours (ref.)*	*62.8255 (62.079, 63.5719)*			*0.2848 (0.2695, 0.3)*		*62.82*	*64.82*	*71.94*	*78.49*	

a Figures in parentheses are 95% confidence intervals; b. Reference category rows are in italics.

### The Unconditional Growth Model

The unconditional growth model showed that at a starting age benchmarked to 50 months, the initial PPVT score was 62.88, with a growth rate from the period 50 to 119 months of 0.29 PPVT points per month (p = <0.0001). This model has an overall fit (pseudo R^2^) of 0.53. This showed that more than half of the variance in receptive vocabulary growth was explained by time (i.e., age).

### Conditional Growth Models

#### Bivariate growth modelling

We tested bivariate associations with PPVT intercept and slope for each of the 8 child, 10 maternal, and 10 family variables in a series of conditional growth models (see Table 3).

For the child variables, most bivariate effects with PPVT were in the small to moderate range. The group of children performing in the lowest quintile on the *Who am I?* test of school readiness, exhibited an effect size bordering on large (*d*_i_ = 0.73). These children with low school readiness had PPVT performances that started 4.5 points, or 14.5 months lower relative to those children in the highest *Who am I?* quintile. However, their onward vocabulary growth differential was among the highest observed (0.037; *d*_s_ = 0.30) allowing these children to halve this initial gap to 2.5 PPVT points, representing a subsequent decrement of about 8 months in vocabulary growth at 105 months relative to the group in the highest *Who am I?* quintile. Within the quintile categories of the *Who am I?* there were differential effects on both the PPVT intercept and the slope. Progressively poorer *Who am I?* performances were associated with progressively poorer PPVT initial vocabulary performance. However, those children in the lowest two quintile groups on the *Who am I?* were the ones that exhibited more rapid growth in onward vocabulary over time relative to the children in the highest quintile group.

Among the child variables the candidate predictors for persistent and reactive temperament and Aboriginal status showed the next highest effects on vocabulary (*d*_i_ = 0.42, 0.41. 0.40 respectively). The PPVT performance of children with low persistence started about 2.6 PPVT points or 8.8 months lower than those children with high persistence. The difference in the rate of change in vocabulary for those children with low persistence was essentially negligible and they were about 6.3 months behind in their PPVT performance 105 months later relative to children with high persistence. A similar pattern was evident in those children with high levels of temperamental reactivity. These children started about 2.5 PPVT points or 8.5 months lower than those children with low reactivity. These children evinced slightly more positive vocabulary change over time relative to those children with low reactivity, but none-the-less remained about 4.7 months behind at 105 months. We would note here that the third measure of temperament – sociability – showed weaker associations with initial vocabulary performance (*d*_i_ = 0.22). Relative to children with high sociability, those with low sociability started about 1.4 PPVT points or 4.8 months lower. Their rate of vocabulary change was not different from children with high sociability (*d*_s_ = 0.03) and they were about 1.2 PPVT points, or 4.1 months behind in their vocabulary performance at 105 months.

Aboriginal children started about 2.5 PPVT points, or 8.5 months below non-Aboriginal children (*d*_i_ = 0.40) and displayed a negligible difference in growth rate (*d*_s_ = 0.05) over the period to 105 months maintaining this initial difference in vocabulary to finish 2.2 PPVT points or 7.4 months behind non-Aboriginal children.

Smaller effects on the vocabulary intercept were observed for low birthweight status (*d*_i_ = 0.34). These children started about 2.1 PPVT points lower, or 7 months behind children with normal birthweight. This initial difference in PPVT vocabulary performance was approximately halved over time by a slightly more rapid change in vocabulary over time (*d*_s_ = 0.14) in the low birthweight group and their performance was about 1.2 PPVT points, or 3.9 months behind at 105 months relative to normal birthweight children.

Finally, we found negligible effects on the PPVT intercept and slope for both gender and ear infections.

There were 10 maternal predictor variables assessed at this stage. In relative terms, the largest effect size on the vocabulary intercept was for maternal parenting consistency (*d*_i_ = 0.55). That is, a medium effect size. Children who experienced a low level of parenting consistency started 3.5 PPVT points, or about 11.4 months behind in their vocabulary performance relative to those children who experienced high levels of parenting consistency. The rate of change of their PPVT over time was the largest among the quintiles of parenting consistency (*d*_s_ = 0.22), and by 105 months they had almost halved the initial PPVT gap to about 2 points, or 6.5 months lower than those children who experienced high levels of parenting consistency. As levels of parenting consistency declined, so too did the PPVT intercept.

The effect of maternal inductive reasoning at intercept was less marked (*d*_i_ = 0.20), with children who experienced a low level of maternal inductive reasoning starting 1.3 PPVT points, or about 4.2 months behind in their vocabulary performance relative to those children who experienced high levels of maternal inductive reasoning. The growth differential was negligible (0.006, *d*_s_ = 0.05), with the initial PPVT gap persisting over time.

Maternal education showed predominant intercept effects. Relative to mothers with post-school education, the PPVT intercept of those children whose mothers had Year 11 or lower levels of education was 3 points or 10.3 months lower (*d*_i_ = 0.48). The rate of change in the PPVT performance was negligible (*d*_i_ = 0.04) and by 105 months their PPVT performance was 2.7 points or 9.4 months lower than children whose mothers had post-school education. The overall effect of changing levels of maternal education was confined to the PPVT initial performance (i.e., the intercept) rather than on producing onward change in PPVT over time (i.e. the slope).

Poor maternal mental health was associated with lower initial PPVT performance (*d*_i_ = 0.42). Where mothers reported symptomatic mental health distress their children's initial PPVT performance was 2.6 points, or about 8.4 months lower than the performance of children whose mothers were not classified as symptomatically distressed. Over time the children whose mothers were mentally distressed showed more rapid change in their PPVT score relative to those whose mothers were not distressed (*d*_s_ = 0.24) and at 105 months their PPVT score was about 1 point, or 3.1 months lower.

The effect of maternal employment on PPVT performance was small and observed in children whose mothers were not working relative to children whose mothers were working part-time. Children whose mothers were not working started about 2.1 PPVT points, or about 7 months behind in their vocabulary performance relative to those children whose mothers were working part-time. This initial difference was almost halved (*d*_s_ = 0.15) and their PPVT score at 105 months was about 1 point, or 3.5 months lower than those children whose mother was working part time. The PPVT performances of children whose mothers were working full-time were indistinguishable from those children whose mothers were working part-time.

With respect to maternal age at birth, the only effect that was notable was a small effect (*d*_i_ = 0.32) for the children whose mothers were teenagers at the time of the birth of the child. Relative to the children of older mothers, the initial PPVT performance of children whose mothers were teens was about 2 points, or 6.7 months lower. The rate of change in their PPVT performance was negligible (d_s_ = 0.05) and at 105 months they remained 1.6 points, or 5.6 months behind the children of mothers aged 20–39 years. The PPVT performances of children of mothers aged 20–39 years was not different from children with mothers aged over 40 years at birth.

The effect of maternal smoking on PPVT performance was small (*d*_i_ = 0.25), with the children of smokers starting 1.6 PPVT points or 5.3 months behind at intercept The growth differential was negligible (*d*_s_ = 0.04), with the initial PPVT gap persisting over time.

Finally, the effects on child PPVT performances of maternal alcohol consumption, were very small and inconsistent. So too were the effects of low maternal warmth and high hostility.

Among the family variables measured, reading to the study child showed the single largest effect on the PPVT intercept. Relative to children who were read to daily, diminishing PPVT performances were associated with lower levels of parental reading to the child. So, relative to those children who were read to daily, the PPVT scores of children who were not read to at all was 6.1 points, or 18.6 months, lower (*d*_i_ = 0.98). Importantly, the rate of change in the PPVT performance of children where were not read to was among the largest observed (*d*_s_ = 0.38) allowing them to almost halve the gap between those who received daily reading. By 105 months their PPVT performance was 3.5 points, or 10.6 months lower than those children who were read to daily.

A large effect size was also observed on the initial PPVT performance of NESB children (*d*_i_ = 0.74). The PPVT scores of NESB children were 4.6 points, or 16.3 months lower than the performance of children from English speaking households. The results for NESB children also showed the largest effect size observed for change in PPVT performance over time (*d*_s_ = 0.49). Thus the 16.3 month gap between their PPVT performance relative to English speaking children was reduced to 1.3 PPVT points, or 4.5 months at 105 months.

Large effects were also observed on initial PPVT performance for low family income and increasing family size. Relative to families earning $2000 per week, the initial PPVT performance of children in families earning $600 per week was 4.3 points, or 13.9 months lower (*d*_i_ = 0.69) and by 105 months the rate of change in their PPVT score (*d*_s_ = 0.24) had reduced this gap to about 2.7 PPVT points, or 8.5 months.

This same pattern of effects was paralleled with respect to family size. Relative to only children, the initial PPVT performance of those children who had four or more siblings was 3.9 points, or 12.7 months lower (*d*_i_ = 0.63). We observed linear effects on the PPVT intercept with decreasing PPVT performance as the number of siblings increased. These effects appeared confined to the intercept with the PPVT performances remaining in relative position over the period to 105 months. There were no differential slope effects.

Small effects were observed for financial distress as measured by the need for an Australian Health Care Card, area disadvantage (SEIFA), financial hardship and single mother status.

The PPVT initial performance of children in families needing a Health Care Card was 2.2 points or 7.5 months lower than the performance of children in families that did not need a Health Care Card (*d*_i_ = 0.36). At 105 months these children were 1.7 points or 5.8 months lower in their PPVT performance with no differential rate of change (*d*_s_ = 0.08).

The initial PPVT performance of children living in the areas of high social and economic disadvantage (i.e. lowest SEIFA quintile) was about 2 points or 6.8 months lower than those children living in the highest SEIFA quintile. Moreover, area disadvantage had a negative effect on PPVT differential, with children in the most disadvantaged areas falling further behind (*d*_s_ = 0.11) such that by 105 months these children had a PPVT score 2.7 points or 9.5 months lower.

Financial hardship and sole mother status were near the threshold for small effects on initial PPVT-III scores (both *d*_i_ = 0.29). Both showed virtually identical effects on initial PPVT scores: Children in families with financial hardship had initial PPVT scores about 1.8 points or 6 months lower than in families without hardship. There was a negligible rate of change in PPVT performance by 105 months and these children's scores were 1.1 points and 3.7 months lower. The results for children of sole mother families were the same.

Finally the effects of playgroup attendance and hours of week in care on initial PPVT scores and on the rate of PPVT growth were negligible.

#### Multivariate growth modelling

Table 4 contains the 16 predictor variables with initial effects *d*> = 0.30 selected for multivariate modelling. We tested independent associations with PPVT intercept and slope for each of the 5 child, 5 maternal and 6 family variables. This model has an overall fit (pseudo R^2^) of 0.6082. Thus the predictors accounted for around an additional 7% of the variance beyond the child's age, compared with the unconditional growth model (pseudo R^2^ of 0.53). While the age of the child was, overwhelmingly, the most important predictor of receptive vocabulary growth, the effects of other predictors with an effect size of d> = 0.3 equated to a 6 month difference in receptive vocabulary at the intercept.

**Table 4:** Multivariate associations between child, maternal and family characteristics and receptive vocabulary growth 4–8 years.a,b

Variables	Initial effect			Growth rate		PPVT scores at 50, 57, 82 and 105 months				Difference @105 months
	**Intercept**	**Cohen's ** ***d*** ** (** ***d*** **_i_)**	**Months**	**Slope**	**Cohen's ** ***d*** ** (** ***d*** **_s_)**	**50**	**57**	**82**	**105**	**PPVT points/Months**
**Overall reference group**	*73.2646 (72.078, 74.4512)*			*0.2094 (0.1823, 0.2364)*		*73.26*	*74.73*	*79.97*	*84.78*	
***Child variables***										
**Ethnicity**										
SC ATSI	−0.1948 (−1.3386, 0.949) (n.s.)	−0.03 (−0.21, 0.15)	−0.94	−0.00271 (−0.02943, 0.02401) (n.s.)	−0.02 (−0.24,0.19)	73.07	74.52	79.68	84.44	−0.34 (−1.66)
**Birthweight**										
Low birthweight	−1.386 (−2.1286, −0.6434)	−0.22 (−0.34, −0.1)	−6.36	0.008674 (−0.00825, 0.0256) (n.s.)	0.07 (−0.07,0.21)	71.88	73.41	78.86	83.87	−0.91 (−4.17)
**Who Am I?**										
quintile 1 (lowest)	−3.634 (−4.2233,−3.0448)	−0.58 (−0.68, −0.49)	−14.65	0.0387 (0.02523, 0.05218)	0.31 (0.2, 0.42)	69.63	71.37	77.57	83.28	−1.51 (−6.07)
quintile 2	−2.1187 (−2.661,−1.5765)	−0.34 (−0.43, −0.25)	−9.23	0.02017 (0.007877,0.03247)	0.16 (0.06, 0.26)	71.15	72.75	78.49	83.77	−1.01 (−4.40)
quintile 3	−1.2235 (−1.804,−0.6431)	−0.2 (−0.29, −0.1)	−5.61	0.008677 (−0.00447, 0.02183) (n.s.)	0.07 (−0.04,0.17)	72.04	73.57	79.02	84.04	−0.75 (−3.42)
quintile 4	−0.75 (−1.2948,−0.2052)	−0.12 (−0.21, −0.03)	−3.54	0.002629 (−0.00967, 0.01493) (n.s.)	0.02 (−0.08,0.12)	72.51	74.00	79.30	84.18	−0.61 (−2.86)
*quintile 5(ref.)*										
**Persistence**										
Quintile 1 (lowest persistence)	−0.5205 (−1.1595, 0.1184)	−0.08 (−0.19, 0.02)	−2.48	0.000222 (−0.0144, 0.01484) (n.s.)	0 (−0.12,0.12)	72.74	74.21	79.45	84.27	−0.51 (−2.42)
quintile 2	−0.4589 (−1.0408, 0.1231) (n.s.)	−0.07 (−0.17, 0.02)	−2.17	0.0018 (−0.0114, 0.015) (n.s.)	0.01 (−0.09,0.12)	72.81	74.28	79.56	84.42	−0.36 (−1.70)
quintile 3	0.02473 (−0.5608, 0.6103) (n.s.)	0 (−0.09, 0.1)	0.12	−0.00265 (−0.01597, 0.01067) (n.s.)	−0.02 (−0.13,0.09)	73.29	74.74	79.91	84.66	−0.12 (−0.59)
quintile 4	0.07506 (−0.5378, 0.6879) (n.s.)	0.01 (−0.09, 0.11)	0.37	−0.00751 (−0.02146, 0.00645) (n.s.)	−0.06 (−0.17,0.05)	73.34	74.75	79.80	84.44	−0.34 (−1.67)
*quintile 5 (ref.)*										
**Reactivity**										
quintile 1 (most reactive)	−0.7519 (−1.3411,−0.1627)	−0.12 (−0.21, −0.03)	−3.47	0.007057 (−0.00639, 0.0205) (n.s.)	0.06 (−0.05,0.16)	72.51	74.03	79.44	84.42	−0.36 (−1.68)
quintile 2	−0.1674 (−0.7573, 0.4224) (n.s.)	−0.03 (−0.12, 0.07)	−0.80	0.000588 (−0.01292, 0.01409) (n.s.)	0 (−0.1, 0.11)	73.10	74.57	79.82	84.65	−0.14 (−0.64)
quintile 3	−0.4307 (−0.9946, 0.1332) (n.s.)	−0.07 (−0.16, 0.02)	−1.99	0.006924 (−0.00595, 0.0198) (n.s.)	0.06 (−0.05,0.16)	72.83	74.35	79.76	84.73	−0.05 (−0.23)
quintile 4	−0.2915 (−0.8423, 0.2594) (n.s.)	−0.05 (−0.13, 0.04)	−1.38	0.001913 (−0.01065, 0.01448) (n.s.)	0.02 (−0.09,0.12)	72.97	74.45	79.74	84.60	−0.19 (−0.88)
*quintile 5 (ref.)*										
***Maternal variables***										
**Mothers Age at birth**										
Teen	−0.8424 (−2.0259, 0.3411) (n.s.)	−0.13 (−0.32, 0.05)	−3.94	0.004293 (−0.0232, 0.03179) (n.s.)	0.03 (−0.19,0.25)	72.42	73.92	79.26	84.18	−0.61 (−2.84)
40+	0.6865 (−0.3546, 1.7275) (n.s.)	0.11 (−0.06, 0.28)	3.31	−0.00207 (−0.02593, 0.02179) (n.s.)	−0.02 (−0.21,0.17)	73.95	75.40	80.59	85.35	0.57 (2.76)
**Mother K6 symptomatic**										
Yes	−1.097 (−1.6039,−0.5901)	−0.18 (−0.26, −0.09)	−4.81	0.01857 (0.00696,0.03018)	0.15 (0.06, 0.24)	72.17	73.76	79.46	84.71	−0.08 (−0.33)
**Maternal education**										
Year 12	−0.8962 (−1.3596,−0.4328)	−0.14 (−0.22, −0.07)	−4.24	0.002166 (−0.00836, 0.0127) (n.s.)	0.02 (−0.07, 0.1)	72.37	73.85	79.14	84.00	−0.78 (−3.67)
Year 11 or less	−1.2802 (−1.7719,−0.7885)	−0.21 (−0.28, −0.13)	−6.14	−0.00105 (−0.01223, 0.01014) (n.s.)	−0.01 (−0.1, 0.08)	71.98	73.44	78.65	83.44	−1.34 (−6.42)
*University (ref.)*										
**Maternal work hours**										
zero hours (includes not in labour force etc.)	−0.06478 (−0.4619, 0.3323) (n.s.)	−0.01 (−0.07, 0.05)	−0.30	0.00454 (−0.00453, 0.01361) (n.s.)	0.04 (−0.04,0.11)	73.20	74.70	80.05	84.97	0.18 (0.86)
full-time: 38 hours +	−0.3069 (−0.8803, 0.2665) (n.s.)	−0.05 (−0.14, 0.04)	−1.44	0.003863 (−0.00916, 0.01689) (n.s.)	0.03 (−0.07 0.14)	72.96	74.45	79.78	84.69	−0.09 (−0.44)
*part-time: 1-37 hours (ref.)*										
**Maternal consistency**										
quintile 1 (least consistency)	−1.0598 (−1.6547,−0.4648)	−0.17 (−0.27, −0.07)	−4.79	0.01173 (−0.00185, 0.02532) (n.s.)	0.09 (−0.01, 0.2)	72.20	73.75	79.28	84.37	−0.41 (−1.88)
quintile 2	−0.3416 (−0.9466, 0.2633) (n.s.)	−0.05 (−0.15, 0.04)	−1.62	0.001323 (−0.01248, 0.01512) (n.s.)	0.01 (−0.1, 0.12)	72.92	74.40	79.67	84.51	−0.27 (−1.28)
quintile 3	−0.3585 (−0.8938, 0.1768) (n.s.)	−0.06 (−0.14, 0.03)	−1.62	0.01184 (−0.00036, 0.02405) (n.s.)	0.09 (0, 0.19)	72.91	74.45	79.99	85.07	0.29 (1.32)
										
quintile 4	0.1326 (−0.3986, 0.6638) (n.s.)	0.02 (−0.06, 0.11)	0.63	0.000184 (−0.0119, 0.01227) (n.s.)	0 (−0.1, 0.1)	73.40	74.86	80.10	84.92	0.14 (0.68)
*quintile 5 (ref.)*										
***Family variables***										
**Mother Non-English Speaking Background**										
Yes	−4.2284 (−4.7933,−3.6635)	−0.68 (−0.77, −0.59)	−15.81	0.058 (0.04502, 0.07098)	0.46 (0.36, 0.57)	69.04	70.91	77.59	83.74	−1.04 (−3.88)
**Siblings**										
One	−0.275 (−0.8864, 0.3364) (n.s.)	−0.04 (−0.14, 0.05)	−1.37	−0.00905 (−0.02303,0.00494) (n.s.)	−0.07 (−0.18,0.04)	72.99	74.39	79.40	84.01	−0.77 (−3.86)
Two	−0.8326 (−1.4946,−0.1706)	−0.13 (−0.24, −0.03)	−4.13	−0.00774 (−0.02285,0.00736) (n.s.)	−0.06 (−0.18,0.06)	72.43	73.84	78.89	83.52	−1.26 (−6.24)
Three	−1.7821 (−2.6137,−0.9505)	−0.29 (−0.42, −0.15)	−8.03	0.01255 (−0.00661, 0.03171) (n.s.)	0.1 (−0.05,0.25)	71.48	73.04	78.58	83.69	−1.09 (−4.92)
four plus	−2.2059 (−3.3696,−1.0422)	−0.35 (−0.54, −0.17)	−10.09	0.009125 (−0.01798, 0.03623) (n.s.)	0.07 (−0.14,0.29)	71.06	72.59	78.05	83.08	−1.70 (−7.80)
*zero (ref.)*										
**Family income per week**										
Under $600	−1.6085 (−2.3946,−0.8223)	−0.26 (−0.38, −0.13)	−7.13	0.01618 (−0.00189, 0.03424) (n.s.)	0.13 (−0.02,0.27)	71.66	73.24	78.87	84.06	−0.72 (−3.19)
$600–$999	−0.8669 (−1.5007,−0.2332)	−0.14 (−0.24, −0.04)	−4.07	0.003574 (−0.01086, 0.01801) (n.s.)	0.03 (−0.09,0.14)	72.40	73.89	79.21	84.11	−0.67 (−3.15)
$1000–$1499	−0.3445 (−0.9206, 0.2315) (n.s.)	−0.06 (−0.15, 0.04)	−1.63	0.001839 (−0.01125, 0.01493) (n.s.)	0.01 (−0.09, 0.12)	72.92	74.40	79.68	84.54	−0.24 (−1.15)
$1500–$1999	−0.3736 (−0.9753, 0.2282) (n.s.)	−0.06 (−0.16, 0.04)	−1.74	0.005578 (−0.00809, 0.01924) (n.s.)	0.04 (−0.06, 0.15)	72.89	74.40	79.77	84.71	−0.07 (−0.31)
*$2000 or more (ref.)*										
**Health care card**										
Yes	0.1919 (−0.3539, 0.7377) (n.s.)	0.03 (−0.06, 0.12)	0.95	−0.00649 (−0.01913, 0.00614)	−0.05 (−0.15,0.05)	73.46	74.88	79.95	84.62	−0.17 (−0.81)
**SEIFA disadvantage index**										
quintile 1 (lowest SEIFA)	−0.05467 (−0.6572, 0.5479) (n.s.)	−0.01 (−0.11, 0.09)	−0.30	−0.0261 (−0.0398, −0.01241)	−0.21 (−0.32, −0.1)	73.21	74.49	79.08	83.29	−1.49 (−8.13)
quintile 2	0.232 (−0.358, 0.8221) (n.s.)	0.04 (−0.06, 0.13)	1.29	−0.02955 (−0.04295,−0.01614)	−0.24 (−0.34, −0.13)	73.50	74.76	79.25	83.39	−1.3 (−7.75)
quintile 3	−0.2224 (−0.8006, 0.3558) (n.s.)	−0.04 (−0.13, 0.06)	−1.12	−0.0111 (−0.02425,0.00205) (n.s.)	−0.09 (−0.19,0.02)	73.04	74.43	79.39	83.95	−0.83 (−4.20)
quintile 4	0.1137 (−0.4669, 0.6943) (n.s.)	0.02 (−0.07, 0.11)	0.58	−0.0136 (−0.02679,−0.00042)	−0.11 (−0.21, 0)	73.38	74.75	79.64	84.15	−0.63 (−3.24)
*quintile 5 (ref.)*										
**Reads to child**										
Not at all	−2.8167 (−3.974,−1.6594)	−0.45 (−0.64, −0.27)	−12.13	0.02282 (−0.00372, 0.04936) (n.s.)	0.18 (−0.03, 0.4)	70.45	72.07	77.88	83.22	−1.56 (−6.72)
1–2 days/week	−1.4339 (−1.9471,−0.9207)	−0.23 (−0.31, −0.15)	−6.62	0.007249 (−0.00441, 0.01891) (n.s.)	0.06 (−0.04,0.15)	71.83	73.35	78.76	83.75	−1.04 (−4.78)
3–5 days/week	−0.9475 (−1.3678,−0.5272)	−0.15 (−0.22, −0.08)	−4.39	0.00642 (−0.00316, 0.016) (n.s.)	0.05 (−0.03,0.13)	72.32	73.83	79.22	84.19	−0.59 (−2.75)
*Daily (ref.)*										

a Figures in parentheses are 95% confidence intervals; b. Reference category rows are in italics.

Of the sixteen variables in the multivariate model, NESB and *Who am I?* showed medium effects on initial adjusted PPVT performance (*d*_i_ = 0.68 and *d*_i_ = 058 respectively) and reading to the child and number of siblings showed small effect sizes on initial adjusted PPVT performance (*d*_i_ = 0.45 and *d*_i_ = 0.35 respectively).

In the multivariate model, NESB showed the largest effect on initial PPVT performance with PPVT scores 4.2 points or about 16 months behind families who were of English speaking status. In the multivariate model, this initial gap in PPVT performance had closed to 1.0 point or 3.9 months at 105 months.

Poor performances on the *Who am I?* showed effects on the initial PPVT performance, evident also in the performances of children at 105 months. Relative to children in the highest quintile of the *Who am I?*, those in the lowest quintile had initial PPVT scores 3.6 points or about 15 months lower. The rate of change in the PPVT score for those children in the lowest quintile of *Who am I?* narrowed the initial gap in performance to 1.5 PPVT points or 6.1 months.

The effects of daily reading to the child remained evident but reduced in the multivariate model. These effects were confined to the initial PPVT performance (i.e. the intercept) and resulted in children maintaining relative position with respect to PPVT performance at 105 months. When compared with those children who were read to daily, the initial PPVT performance of those children who were not read to at all was 2.8 points or 12.1 months lower. The rate of change in PPVT was essentially negligible for all levels of reading from the initial measure to the measure at 105 months. So, by 105 months the group of children who were reported as not being read to at the Wave 1 interview were 1.6 PPVT points, or 6.7 months lower than those who had been read to daily.

Sibling effects, while small, were apparent – particularly for those children with 2, 3 or 4 siblings, these effects being confined to the initial performance (i.e., intercept). So for example, relative to those children with no siblings, the initial PPVT performance for those with 4 siblings was 2.2 points or about 10 months lower. There were no apparent differential effects in the rate of PPVT change over time and these children were 1.7 points or 7.8 months lower at 105 months.

Of the remaining eight predictors, income, low birthweight and maternal education showed the next largest effects (*d*_i_ = 0.26, 0.22 0.21 respectively). These variables demonstrated effects on PPVT initial performance (i.e. intercept). There were no differential rates of change in PPVT performance over time associated with income and mothers education. Relative to children in high income families, the initial PPVT performance of those in low income families was 1.6 points or 7.1 months lower and at 105 months this gap had diminished to 0.7 adjusted PPVT points, or 3.2 months. Low birthweight children started 1.4 PPVT points or 6.4 months lower, relative to normal birthweight children. The growth rate differential was negligible (*d*_s_ = 0.07) and by 105 months the gap remained 0.9 PPVT points or 4.2 months. For children in families with mothers who had 11 or less years of school, their initial PPVT score was 1.3 points or about 6 months lower relative to children from families where mother had some level of post-school education. No differential rates of change in PPVT scores was observed over time, and this group persisted with relatively lower PPVT performance with PPVT scores of 1.3 points or about 6 months at 105 months.

After accounting for the other variables in the multivariate model, there was no independent effect of area disadvantage on initial PPVT, with the initial PPVT of children living in the areas of high social and economic disadvantage (i.e. lowest SEIFA quintile) about 0.05 points or 0.2 months lower than those children living in the highest SEIFA quintile. However, while the effect on the intercept was negligible, the negative receptive vocabulary growth differential associated with these areas (D_s_ = 0.21) persisted, so by 105 months children in the areas with the greatest social and economic disadvantage were 1.5 PPVT points or 8.1 months behind.

Finally, effects for Aboriginal status, temperament variables, maternal age, maternal mental health, maternal work hours, parenting consistency, and Health Care Card status showed extremely small and inconsistent effects well below criterion threshold (i.e., d = >0.30).

## Discussion

The aims of this study were to investigate trajectories of receptive vocabulary growth from 4–8 years and to identify child, maternal and family predictors of variation in the intercept and slope of these trajectories.

The results showed substantial variation in receptive vocabulary ability at 4, 6 and 8 years as well as in the rate of growth between 4–8 years. These results mirror studies of the onset and growth of receptive and expressive vocabulary development in the first three years of life [60], [61]. This suggests that receptive vocabulary acquisition continues to be characterised by variability in the preschool and school years and is not just a pattern observed in very young children at the onset of language acquisition. This pattern of variability rather than convergence over time is not surprising given that receptive vocabulary growth does not have a developmental endpoint, unlike the acquisition of speech sounds or grammar.

### Bivariate and Multivariate Growth Model Effects for Receptive Vocabulary

The child, maternal and family variables that were included in both the bivariate and multivariate models were those with a bivariate effect size d> = 0.30 (see Table 5). The child characteristics in both models were ethnicity, birthweight, school readiness and temperament. The maternal characteristics in both models were age, mental health distress, education, work hours, and parenting consistency. The family characteristics in both models were NESB, sibship size, income, health care card, socio-economic area disadvantage and book reading. Once multivariately adjusted, the child, maternal and family risk factors for low receptive vocabulary ability at four years were low birthweight, low school readiness, teenage motherhood, mental health distress, low maternal education, low maternal consistency, NESB, one or more siblings, low income, health care card, socio-economic area disadvantage and book reading. Except for socio-economic disadvantage, these risk factors were associated with a positive growth differential between 4 – 8 years. The positive growth differential associated with low birthweight, low school readiness, teenage motherhood, mental health distress, low maternal education, low maternal consistency, NESB, one or more siblings, low income, health care card, and book reading, meant that children with these risk factors narrowed the gap in receptive vocabulary ability at the intercept, to varying degrees (see Table 5).

The adjusted effects are important to understand with respect to multiple risk. For most predictors, adjusted effect sizes are negligible to small. The total amount of variance that the predictors account for is an additional 7% after adjusting for advancing age, which accounts for another 52% of the variance in vocabulary development. This 7% is a surprisingly small percentage of increase in variance accounted for over and above that accounted for by age. This is consistent with other population level studies that have reported that child, maternal and family characteristics explain little of the variation observed in young children's language acquisition [62]. These adjusted estimates provide an indication of the independent contribution that the predictors make so it is important to remember that risks occur multiply and that the adjusted effect sizes are in this sense additive.

Based on effect sizes, this study failed to reveal strong explanatory factors for variable growth in children's receptive vocabulary abilities, apart from the child's age. That is, the effect sizes for risks associated with low receptive vocabulary ability were mostly medium to small. This is entirely consistent with the results of other studies of children's language acquisition that have shown that prediction of variability in language acquisition is modest at best [62], [63], [64]. However, when children's receptive vocabulary scores were equated to months, the impact of adjusted risk factors with medium to small effect sizes equated to noteworthy developmental differences in months of receptive vocabulary growth. For example, the risk for low receptive vocabulary associated with not reading to the study child had a small effect size at intercept (*d* = −0.45). However, this equated to a 12-month gap in receptive vocabulary development for children who were not read to at all, relative to children who were read to daily.

Of particular interest, was the extent to which risks associated with a low intercept at 4 years were also associated with a lower rate of growth from 4–8 years. From an initial set of 28 candidate predictors, 10 risks were associated with intercept effects. These risks in order of magnitude were: Maternal NESB, low school readiness, child not read to at home, four or more siblings, low family income, low birthweight, low maternal education, maternal mental health distress, low maternal parenting consistency and high child temperament reactivity. Notably, none of the risks that influenced the intercept at 4 years were associated with a lower rate of vocabulary growth from 4–8 years. Instead, maternal NESB, low school readiness and maternal mental health distress were associated with a higher rate of growth, although not sufficient to close the receptive vocabulary gap for children with and without these risks at 8 years. Male gender had negligible effects on receptive vocabulary ability at intercept and slope. The results of meta-analyses concur with the results of the present study, that gender differences in language ability are negligible [59]. Convergent evidence for negligible gender differences in language ability is that the prevalence of language impairment in the general population is 8% for boys and 6% for girls and not significantly different [65].

The only risk factor associated with a slower rate of growth was socioeconomic area disadvantage. At 8 years, the gap between children with and without area disadvantage was equivalent to eight months of receptive vocabulary growth. Thus the gap between children with and without area disadvantage widened in the early years of school. The higher rate of growth for children with maternal NESB and maternal mental health distress narrowed the receptive vocabulary gap at 8 years to less than 4 months. The higher rate of growth for children with low school readiness between 4–8 years more than halved the gap of 15 months at 4 years compared to a six-month gap at 8 years. The remaining risks associated with lower receptive vocabulary ability at 4 years; low birthweight, low maternal education, low family income, not read to at home, four or more siblings, low maternal parenting consistency and high child temperament reactivity were not associated with higher or lower receptive vocabulary growth from 4–8 years. For children with these risk factors, the receptive vocabulary gaps apparent at 4 years were still evident at 8 years. These results showed that receptive vocabulary disparities evident before the start of formal school persisted over time, although the extent of the disparities at 8 years varied across risk types. The largest gap in receptive vocabulary abilities at 8 years was between children with and without socio-economic area disadvantage. The concerning pattern of change over time for these children was slower growth and increasing disparity. This is consistent with the findings from a study of 10,366 children in the U.S. Children of the National Longitudinal Survey of Youth 1979 (CNLSY). The study used growth curve models to investigate social class and race differences in receptive vocabulary growth trajectories from 3 – 13 years. The highest rate of receptive vocabulary growth was in the preschool years and social class and race disparities in receptive vocabulary abilities that were evident in the preschool years did not close (or widen) in the school years. Their findings pointed towards the preschool years as the critical time for intervention because this was the period of highest growth and also the period when disparities emerged [28]. Even with pre-school education between 3–5 years, the SES-gap in children's language abilities has been shown to persist. While pre-school education did not close the gap, the SES-gap widened for low-SES children who did not participate in pre-school education [37].

### Implications for Prevention and Intervention

With important exceptions, these being low school readiness and socio-economic area disadvantage, none of the remaining predictors would suggest that a preventive strategy or intervention to address vocabulary development (or language development more widely) should focus on a single risk exposure. Instead, the pattern of weak adjusted effect sizes would suggest a broad-based or universal “scaffold” of developmental opportunity and interventions able to address multiple risks would have greater merit. With respect to intensity of intervention, like the predictors, these opportunities and interventions would need to operate close to the child, be available fairly regularly and over an extended period of time. In this regard, we suspect consistency of regular but modest participation in these developmental opportunities is more critical than short, high intensity interventions. The issue to then consider is the extent to which such developmental strategies are adjusted and made universally proportionate (or not) to address risks associated with socioeconomic area disadvantage and low school readiness.

### Future Research

The results of this study showed a mixed picture of risks for low receptive vocabulary ability between 4–8 years. The widest receptive vocabulary gaps between risk exposed and non-risk exposed children were evident at 4 years, and with the exception of socio-economic area disadvantage, the gaps narrowed through the early years of school. As the children in this study progress through the school grades, it will be important for future research will determine: (1) Risk factors for persistently low receptive vocabulary ability from 4 – 8 years; (2) the extent to which low receptive vocabulary ability projects to low literacy ability and (3) and the configuration of risks for low literacy ability.
