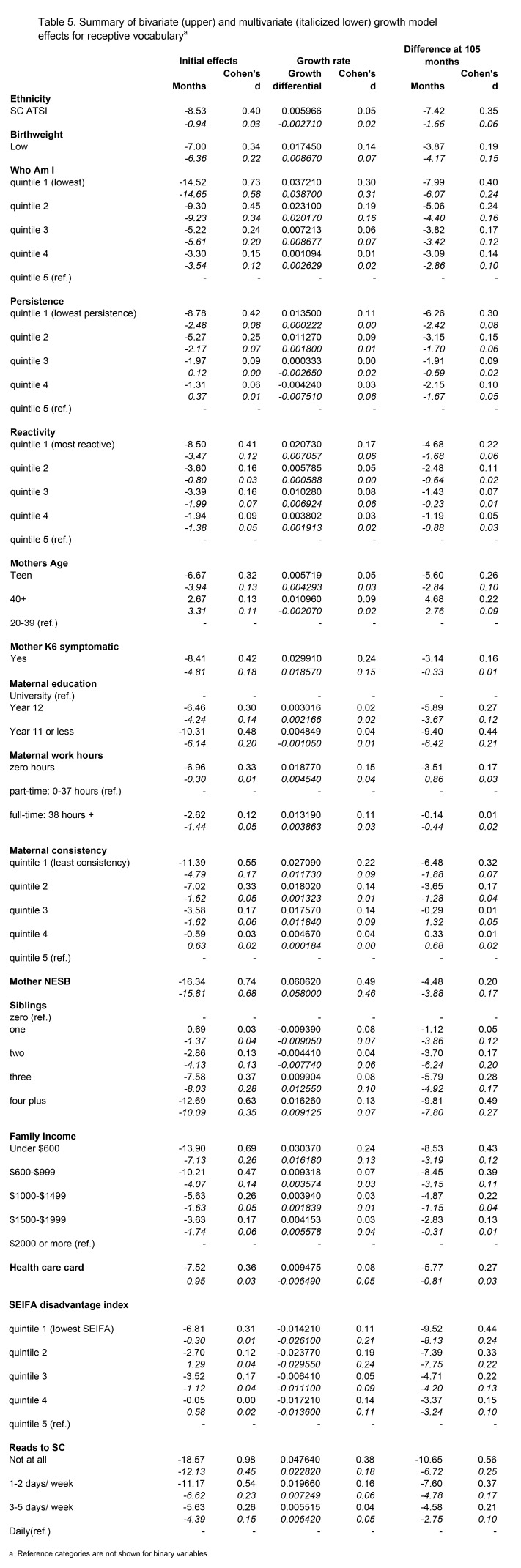# Correction: Risk Factors for Children's Receptive Vocabulary Development from Four to Eight Years in the Longitudinal Study of Australian Children

**DOI:** 10.1371/annotation/a730446c-0150-4079-98ea-95b12e1e3c28

**Published:** 2013-11-05

**Authors:** Catherine L. Taylor, Daniel Christensen, David Lawrence, Francis Mitrou, Stephen R. Zubrick

Table 5, "Summary of bivariate (upper) and multivariate (italicized lower) growth model effects for receptive vocabulary^a^" was erroneously omitted. Please see Table 5 here: 

**Figure:**